# Fecal microbiota transplantation for glaucoma; a potential emerging treatment strategy

**DOI:** 10.1016/j.crmicr.2024.100314

**Published:** 2024-11-13

**Authors:** Rasoul Ebrahimi, Yeganeh Farsi, Seyed Aria Nejadghaderi

**Affiliations:** a School of Medicine, Shahid Beheshti University of Medical Sciences, Tehran, Iran; b HIV/STI Surveillance Research Center, and WHO Collaborating Center for HIV Surveillance, Institute for Futures Studies in Health, Kerman University of Medical Sciences, Kerman, Iran

**Keywords:** Fecal microbiota transplantation, Intraocular pressure, Glaucoma, Microbiota, Retinal diseases

## Abstract

Glaucoma is the primary cause of irreversible blindness globally. Different glaucoma subtypes are identified by their underlying mechanisms, and treatment options differ by its pathogenesis. Current management includes topical medications to lower intraocular pressure and surgical procedures like trabeculoplasty and glaucoma drainage implants. Fecal microbiota transplantation (FMT) is an almost effective and safe treatment option for recurrent Clostridium difficile infection. The relationship between bacterial populations, metabolites, and inflammatory pathways in retinal diseases indicates possible therapeutic strategies. Thus, incorporating host microbiota-based therapies could offer an additional treatment option for glaucoma patients. Here, we propose that combining FMT with standard glaucoma treatments may benefit those affected by this condition. Also, the potential safety, efficacy, cost-effectiveness and clinical applications are discussed.

## Highlights

• Glaucoma remains the leading cause of irreversible blindness worldwide, highlighting the need for novel therapeutic strategies.
• Current treatment options for glaucoma focus on lowering intraocular pressure through topical medications and surgical interventions.
• Fecal microbiota transplantation (FMT), initially approved for recurrent Clostridium difficile infection, emerges as a potential adjunctive therapy for glaucoma, targeting bacterial populations and inflammatory pathways.
• Combining FMT with standard glaucoma treatments could enhance therapeutic efficacy, safety, and cost-effectiveness for patients.

## Graphical abstract

**Figure:**
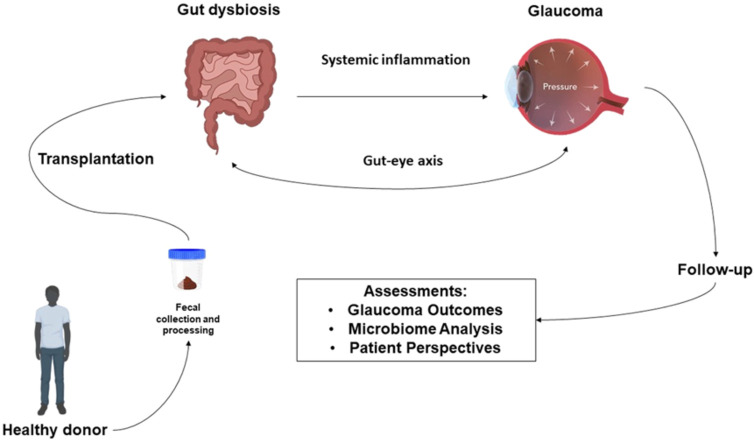

## 1 Introduction

Glaucoma is the foremost cause of permanent blindness globally (Kang and Tanna, 2021). It is estimated that 3.5 % of adults aged 40 to 80 years are affected by glaucoma globally, predicted to surpass 111 million by 2040 (Kang and Tanna, 2021). The increasing pattern of the disease between 1990- 2019, accompanied by a considerable increase in disability-adjusted life years from about 442 to 748 thousands during this period, has made glaucoma a critical concern threatening patients’ vision (Lin et al., 2023).

Glaucoma subtypes are distinguished based on their underlying mechanisms and treatment options are considered regarding the pathogenesis of the disease. Secondary glaucoma, which is less common, is the consequence of another etiology, and treating the underlying pathology would resolve the disease; neovascularization, trauma, certain medications (e.g., corticosteroids), inflammatory conditions, tumors, lens-related problems, and some other ophthalmic conditions such as pigment dispersion or pseudo-exfoliation are the causes of secondary glaucoma (Ekici and Moghimi, 2023; Liu et al., 2023). On the other hand, in primary forms of glaucoma, the primary pathology is either the blockade of the Schlemm's canal (angle closure) or dysfunction in the trabecular meshwork system (open-angle) (Ekici and Moghimi, 2023).

Topical intraocular pressure-lowering medications and surgical interventions including trabeculoplasty and implanting glaucoma drainage implants are the current management options for glaucoma (Michels and Ivan, 2023); Yet, it is highlightable that these medications and interventions do not cure the disease or stop its process, and only decrease the progression of the disease; thus, improving the diagnostic methods, optimizing the current treatment strategies, and developing treatment strategies with better efficacy has been always of great interest (Wagner et al., 2022). Identifying the risk factors of glaucoma or other factors that can improve the response to therapy or interfere with the disease progression is crucial. Aging, certain ethnicities, obesity, comorbidities, dietary and lifestyle-related risk factors, and the use of some medications have been identified as risk factors for glaucoma so far (Dada et al., 2022). Meanwhile, growing evidence supports the role of gut microbiota dysbiosis in the progression of glaucoma (Hernández-Zulueta et al., 2024).

Microbiota is the combination of microorganisms including bacteria, archaea, fungi, protists, algae, and viruses that live with other organisms colonizing the mucocutaneous surfaces of the body with several beneficial effects on our homeostasis (Berg et al., 2020; Leonard and Toro, 2023). They prevent pathogen colonization, promote human digestion by enzyme production, and regulate immune system activity (Ogunrinola et al., 2020; Rackaityte and Lynch, 2020). Imbalanced changes in microbiota load, type, or diversity, known as dysbiosis, result in immune system reactions, inflammatory, autoimmune, and allergic reactions, infectious diseases, cancers, and cardiovascular diseases (Gilbert et al., 2018; Astudillo-de la Vega et al., 2019; Coker, 2022; Xie et al., 2023; Zaramella et al., 2023). Notably, the microbiome does not affect only local, but also several evidence support their systemic direct effects in various off-site organs. The broad spectrum of gut microbiome impact on various diseases led to the concept of “gut-organ axis” (Saxami et al., 2023). The gut-brain, gut-lung, and gut-skin axes are the best-known examples of the interactions between the gut microbiome and various diseases including Alzheimer's disease, Parkinson's disease, asthma, chronic obstructive pulmonary disease, acute respiratory infections, cystic fibrosis, psoriasis, atopic dermatitis, acne vulgaris, rosacea, alopecia areata, and hidradenitis suppurativa (Enaud et al., 2020; Parker et al., 2020; Manos, 2022; Mohamed et al., 2022; Czarnik et al., 2024). The gut-eye axis is also another subset of these axes, and alterations in the gut microbiome can affect the ocular overall condition (Thakur and Sheppard, 2022). Gut dysbiosis can activate the innate immune system, trigger autoimmune reactions, and reduce IgA secretion. Evidence of gut dysbiosis has been identified in patients with Sjögren's dry eye, diabetic retinopathy, glaucoma, macular degeneration, and infectious keratitis (Moon et al., 2020a, b; Napolitano et al., 2021; Pezzino et al., 2023). Changes in homocysteine metabolism, inflammation, altered metabolite panels, molecular mimicry, activation of lipopolysaccharide-toll-like receptor 4 pathway, and direct dissemination of microbes are the proposed mechanisms justifying the role of dysbiosis in pathogenesis glaucoma, changes in intraocular pressure, and retinal ganglion cell count (Huang et al., 2023).

The variety of mechanisms by which microbiota can affect human health and disease has made it a suitable target for directed interventions (Olteanu et al., 2024). Interventions for restoration the dysbiosis include administration of probiotics, prebiotics, symbiotic, and fecal microbial transplantation (FMT) (Dixit et al., 2021). FMT is transferring the stool from a healthy donor to a patient to restore the altered microbial composition (Porcari et al., 2023). The evidence of dysbiosis among patients with glaucoma is increasing steadily, making dysbiosis a potential novel target to improve treatment outcomes (Gong et al., 2020; Petrillo et al., 2020; Gong et al.,2022; Zhang et al., 2022; Campagnoli et al., 2023). Herein, we aim to describe the role of gut dysbiosis in the pathogenesis of glaucoma and conclude the applicability and current feasibility of FMT as an adjacent treatment option to improve disease progression and disease outcomes.

## 2 Microbiota and glaucoma risk factors

**Fig. 1:**
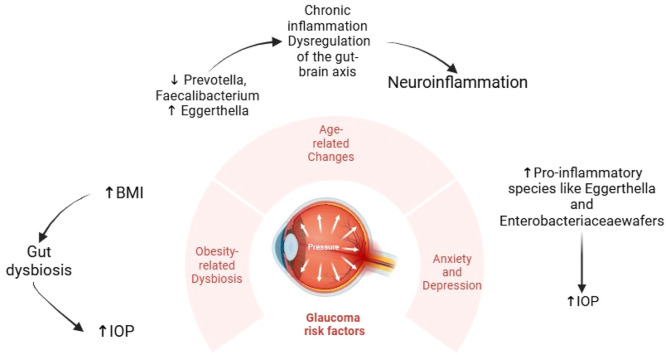
The relationship between microbiota changes and glaucoma risk factors, including age-related changes, obesity, and anxiety/depression. Each peripheral factor highlights specific microbiota alterations and the corresponding effects on inflammatory and metabolic pathways, which may promote neuroinflammation and disease progression in glaucoma patients. IOP: intraocular pressure; BMI: body mass index.

Fig. 1 represents an overview on the relationship between glaucoma risk factors and microbiota changes.

### 2.1 Age-related gut microbiota changes and glaucoma

Aging is a major contributor to glaucoma development and can cause significant changes in the microbiome due to various factors such as inflammation and epigenetic dysregulation. These changes can lead to chronic diseases, metabolic disorders, and impaired gut-brain axis (Cryan et al., 2019; Pellanda et al., 2021), which can further affect the host's behavior and quality of life. Personal lifestyle choices such as diet can also shape the microbiome in the elderly and offer opportunities to make healthy changes (Ghosh et al., 2022). Alterations in the gut microbiota during aging involve a decline in dominant commensal taxa, such as *Prevotella, Faecalibacterium, Eubacterium rectale, Lachnospira, Coprococcus*, and *Bifidobacterium* (Claesson et al., 2012; Jeffery et al., 2016; Ghosh et al., 2020), and an increase in pathogenic microbiota like *Eggerthella, Bilophila*, Fusobacteria, *Streptococcus*, and Enterobacteriaceae (Jeffery et al., 2016; Ghosh et al., 2020). These changes are similar to those seen during infancy when the immune system is immature, highlighting a link between microbiota, aging, and the immune system (Nagpal et al., 2018).

In patients with glaucoma, there are higher levels of proinflammatory cytokines and chemokines and increased intraocular pressure (IOP) (Karlsson et al., 2013). Investigating the association between eye diseases, aging, and the changes in bacterial populations of the microbiota could aid in understanding disease processes and identifying novel interventions (Chen et al., 2023). One approach being investigated for preventing or improving glaucoma and other retinal diseases is the use of probiotics and introducing healthy microbiota (Parolini, 2019). Dysbiosis leads to systemic inflammation and the emergence of many diseases (Floyd and Grant, 2020). Neurodegeneration in glaucoma is linked to inflammation (Rolle et al., 2020). Therefore, the potential role of gut microbiota in retinal disease progression becomes apparent due to dysbiosis and a compromised gut-vessel barrier (Rinninella et al., 2018). Glaucomatous eyes display signs of increased inflammatory signals (Pezzino et al., 2023).

### 2.2 Obesity-related dysbiosis and glaucoma

Obesity and metabolic health may affect the development of glaucoma (Lima-Fontes et al., 2020; Wu et al., 2022). High body mass index (BMI) is associated with decreased choroidal perfusion, ocular blood flow, increased orbital fat, and higher IOP, all of which may negatively contribute to glaucoma development (Klein et al., 1992; Mori et al., 2000; Karadag et al., 2012; Panon et al., 2019; Marshall et al., 2023). The link between obesity and primary open-angle glaucoma (POAG) is complex, with some studies suggesting a higher risk (Jung et al., 2020; Chen et al., 2021b) and others finding no significant association (Pasquale et al., 2010; Na et al., 2020).A recent meta-analysis identified that obesity was a significant risk factor for POAG (Rong and Yu, 2023) and a retrospective multi-central longitudinal analysis determined a correlation between BMI and glaucomatous outcomes. Accordingly, a lower BMI was linked with a higher rate of visual field progression (*P* = 0.01) and an increased likelihood of glaucoma diagnosis (OR, 0.90; 95 % CI, 0.84–0.98). A lower BMI was also correlated with a more unfavorable cross-sectional vertical cup-to-disk ratio (*P* < 0.001) and higher IOP (*P* < 0.001) (Marshall et al., 2023).

Gut microbiome dysbiosis is linked to the development of metabolic diseases such as obesity (Guo et al., 2021). Dysbiosis also contributes to the progression of central nervous system (Pellegrini et al., 2020) and retina degenerative disorders (Scuderi et al., 2021), and also in the development of many ocular diseases (Napolitano et al., 2021; Shivaji, 2021; Xue et al., 2021). Obesity is associated with an elevation in various inflammatory markers, ultimately leading to persistent, low-level inflammation (Artemniak-Wojtowicz et al., 2020). A disturbance in the gut microbiome is associated with this inflammation in people with obesity (Boulangé et al., 2016; Scheithauer et al., 2020). Neuroinflammation is a key element in glaucoma (Mac Nair and Nickells, 2015; Williams et al., 2017), and gut dysbiosis can disrupt immune responses and cause systemic inflammation, which can lead to tissue degeneration and neuroinflammation in the eye. Bacteria and their metabolites can reach the ocular regions through the bloodstream or lymph vessels, causing tissue degeneration and neuroinflammation (Pezzino et al., 2023).

### 2.3 The relationship between anxiety, depression, gut microbiota, and glaucoma

Several population-based studies indicated a link between glaucoma and psychiatric conditions, particularly depression and anxiety (Lin et al., 2010; Zhou et al., 2013; Diniz-Filho et al., 2016; Zhang et al., 2017). Among patients with POAG, depression prevalence ranges from 13 % to 30 %, while anxiety prevalence ranges from 6 % to 25 % (Zhang et al., 2017). Anxiety has been linked to optic disk bleeding, retinal nerve fiber layer loss, and peak IOP, while depression was associated with higher visual field mean deviation (Shin et al., 2021). Moreover, the gut microbiota of depressive and anxious people exhibited numerous pro-inflammatory species like Eggerthella, Enterobacteriaceae, and Desulfovibrio, while short-chain fatty acid (SCFA) producing-species such as Megamonas, Faecalibacterium, Coprococcus, and Clostridium XIVa were less commonly present (Simpson et al., 2021). Aging, obesity, and depression, as glaucoma risk factors, exhibit unique gut SCFA profiles, suggesting that SCFAs may have diverse roles in glaucoma development.

## 3 Mechanisms and implications of gut microbiota in glaucoma

The gut microbiome influences various retinal diseases, including glaucoma, through multiple pathways. Specific taxa within the gut microbiota have a crucial role in this development. For instance, members of the *Lachnospiraceae* family, which produce SCFAs through fermentation (Canani et al., 2011), are generally considered beneficial for human health (Zheng et al., 2021). However, recent studies indicate a possible connection between SCFAs and the onset of glaucoma. Chen et al. found that administering intestinally metabolized SCFAs worsened retinal cell loss. This suggests that intestinal microorganisms and their SCFA metabolites may activate retinal microglia via the microRNA network, leading to neuroinflammatory responses (Chen et al., 2022b). Additionally, another study found that treatment with SCFAs increased microglial activation and exacerbated Parkinson's disease in mouse models (Sampson et al., 2016). A possible hypothesis indicates that the genus *LachnospiraceaeUCG010* is responsible for producing SCFAs, which then helps microglia fully mature and increases their inflammatory potential. Therefore, *LachnospiraceaeUCG010* itself, as well as its SCFA metabolites, may be useful targets for maintaining optic nerve function in glaucoma.

Shibuya et al. investigated the relationship between normal tension glaucoma and a specific variation in the *TLR4* gene, which is responsible for recognizing lipopolysaccharide (LPS) in bacteria (Shibuya et al., 2008). When TLR4 is activated, it triggers the production of inflammatory cytokines and inhibits lipolysis and beta-oxidation, which can limit the growth of bacterial pyruvate and acetyl-CoA. Furthermore, patients with glaucoma exhibit increased TLR4 expression, particularly in retinal microglia cells. However, the relationship between gut microbiome alterations, their pro-inflammatory effects, and TLR4 variations in glaucoma remains unclear. Zinkernagel et al. (Zinkernagel et al., 2017), reported that peripheral injection of bacterial LPS induced axonal degeneration and neuronal loss in two separate glaucoma animal models. This effect was likely due to the upregulation of TLR4 and subsequent activation of complement leading to damage of retinal and optic nerve microglia. To investigate whether bacterial lysates could mitigate glaucoma-induced neurodegeneration, mice in two separate glaucoma models were given low-dose subcutaneous bacterial LPS. This study revealed a connection between peripheral LPS administration and increased activation of optic nerve microglia, contributing to the loss of retinal ganglion cells. Thus, bacteria can activate axonal microglia leading to neurodegeneration.

It has been proposed that intestinal microbiota promotes the production of neuroprotective factors, aiding in the survival of retinal ganglion cells (Gupta, 2015). Additionally, a potential link between glaucoma and Helicobacter pylori (H. pylori) has been proposed since a higher rate of H. pylori infection has been observed in glaucoma patients compared to normal tension controls (Kim et al., 2011). There are several suggested mechanisms for this correlation, including cytokines, ureases, and the neutrophil-activating protein VacA (Alvarez-Arellano and Maldonado-Bernal, 2014). These factors have been shown to cause inflammation and activate immune cells, leading to the differentiation of microglia into phagocytic macrophages within the optic nerve (Neufeld, 1999). Moreover, at the molecular level, epigenetic modifications have also been associated with damage to the optic nerve. These changes in retinal ganglion cells could also be connected to glaucoma alterations. This disrupted homeostasis might be linked to changes in the gut microbiota. Inflammation resulting from microbial dysbiosis could trigger microglial regulation through various pathways, including direct bacterial spread to the optic nerve or retina, bacterial product dissemination, vascular system alterations, and fluctuations in the systemic immune system (Napolitano et al., 2021).

**Fig. 2:**
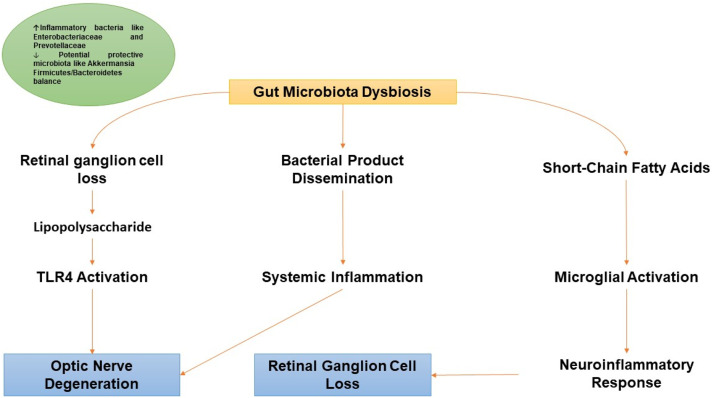
A schematic illustration of how gut microbiota dysbiosis influences glaucoma pathogenesis through pathways involving short-chain fatty acids production, TLR4 activation, immune cell activation, and microbial spread, contributing to neuroinflammation and retinal degeneration.

Additionally, notable differences in gut microbiota between POAG patients and healthy individuals have been observed. Gong et al. found that POAG patients had higher levels of Prevotellaceae, unidentified Enterobacteriaceae, and Escherichia coli, but decreased levels of Megamonas and Bacteroides plebeius compared to healthy individuals (Gong et al., 2020). They noted that certain microbial species were positively or negatively correlated with specific metabolites and clinical phenotypes, suggesting that there are distinct differences in gut microbiota composition between POAG patients and healthy individuals (Gong et al., 2020). The human gut microbiota is mainly composed of Firmicutes and Bacteroidetes bacterial phyla (Frank and Pace, 2008). Reports on changes in the gut microbiota of glaucoma patients vary. In 2014, an association was found between mitochondrial DNA variants *m.15784T>C* and *m.16390G>A* and the Firmicutes and Proteobacteria phyla in glaucoma patients (Ma et al., 2014). Later, Collins et al. found that these variants were enriched in patients with POAG (Collins et al., 2016). When studying mouse models of glaucoma, it was found that when the mice were kept germ-free, there was no neural degeneration associated with glaucoma. However, under specific-pathogen-free conditions, there was a loss of retinal ganglion cells that progressed over time (Chen et al., 2018). Zhang et al. utilized 16S rRNA sequencing and untargeted metabolomic analysis and observed a notable reduction in microbial diversity and distinct variations in bacterial populations in rats with chronic glaucoma in contrast to the controls. Additionally, they observed a distinct shift in gut metabolites (Zhang et al., 2022). DBA/2J mice tend to develop glaucoma between six and eight months of age (John et al., 1998). In comparison to non-glaucoma mice such as C57BL/6J and FVB/NJ mice, these mice had a lower baseline amount of the bacteria Akkermansia and a higher Firmicutes/Bacteroidetes (F/B) ratio in their gut (Ahn et al., 2020). In Wistar rats that had chronically high IOP, there was an increase in the F/B ratio and higher levels of Verrucomicrobia, Romboutsia, and Akkermansia bacteria in their gut. These factors were inversely correlated with the number of retinal ganglion cells. Additionally, a study involving untargeted metabolomics identified 284 metabolites that were expressed differently in these rats, with the metabolism of bile being the main pathway involved (Zhang et al., 2022). The mouse and rat models used to study glaucoma both exhibit a higher F/B ratio in their gut, indicating a similar trend. However, there is a difference in the presence of Akkermansia, which is a protective factor. DBA/2J mice have low levels of Akkermansia while glaucoma rats have high levels of Akkermansia (Huang et al., 2023). Fig. 2 represents a summary of the mechanisms.

## 4 Intraocular and ocular surface microbiota in glaucoma

Multiple studies have documented changes in ocular surface microbiota of glaucoma patients, linking these alterations to the use of anti-glaucoma eye drops (Chang et al., 2022; Priluck et al., 2023). Glaucoma patients who use preserved eye drops have a more diverse microbiome, with more gram-negative bacteria than gram-positive bacteria found in healthy eyes, accompanied by lower levels of tear film and distinctive protein synthesis pathways (Chang et al., 2022). A study analyzing eyelid samples found differences in various taxa, with an increase in *Paenibacillus* and *Dermacoccus* and a decrease in *Morganella* and *Lactococcus* in individuals with uveitis glaucoma (Shin et al., 2022), as well as a greater presence of *Rhodococcus* in uveitis glaucoma samples compared to OAG samples (Lee et al., 2022).

Studies have found differences in the alpha and beta diversity levels of ocular surface microbiota between patients with glaucoma and healthy controls. In glaucoma patients, treated with drops, there is primarily Firmicutes (61.1 %) and Verrucomicrobiota (11.8 %) with decreased Actinobacteriota. Common genus classifications included Akkermansia, Corynebacterium, Faecalibacterium, Lachnospiraceae, and Blautia (Chang et al., 2022). The microbiota of the ocular surface in glaucoma patients contains a higher abundance of anaerobic, gram-negative organisms associated with lipopolysaccharide synthesis and pathways for anaerobic fatty acid synthesis, hydrogen sulfide, and sulfate metabolism, while healthy controls had a higher abundance of gram-positive organisms associated with pathways for carbohydrate synthesis, glycolysis, and oxidative phosphorylation (Chang et al., 2022).

The use of anti-glaucoma eye drops containing benzalkonium chloride as a preservative may significantly alter the types of organisms or microbiota present on the ocular surface (Chang et al., 2022), as benzalkonium chloride suppresses gram-positive organisms. However, a recent study discovered that benzalkonium chloride does not have any discernible impacts on the ocular surface microbiota of glaucoma patients (Priluck et al., 2023). Evidence of bacteria was found in over 1000 human eye samples, challenging the previous belief that the intraocular environment is sterile. It was discovered that P. acnes increased while Staphylococcus warneri decreased in the glaucoma patients (Deng et al., 2021). The discovery of H. pylori in trabeculum and iris specimens from glaucoma patients further supported the involvement of H. pylori infection in the development of POAG (Zavos et al., 2012).

## 5 Oral and gastric microbiota dysbiosis and glaucoma

An imbalanced oral microbiome can impact glaucoma progression. A study found that there is a link between severe periodontal disease and a higher risk of POAG (Pasquale et al., 2016). The presence of H. pylori in the oral microbiome has been linked to both POAG and normal-tension glaucoma (Zeng et al., 2015). Kountouras et al. showed that eradicating H. pylori improved both IOP and visual field defect in individuals who had POAG (Kountouras et al., 2002). The link between H. pylori and glaucoma remains contentious, as another study failed to establish a significant connection between H. pylori and the risk of developing glaucoma (Kurtz et al., 2008).

Oral microbiota dysbiosis can impact the development of glaucoma (Rowan and Taylor, 2018; Mosaddad et al., 2023). The presence of periodontal pathogens leads to chronic inflammation, affecting blood vessels and activating the local immune system in the retina and optic nerve. This allows immune or bacterial components to reach these areas. Subsequently, immune mediators harm optic nerve cells by priming local microglia (Arjunan and Swaminathan, 2022). Patients with glaucoma have a significantly higher oral bacterial load, indicating continuous exposure to elevated bacterial products (Sun et al., 2020; Yoon et al., 2021). Differentially expressed gene analysis revealed a depletion of Lactococcus and an enrichment of Faecalibacterium in patients with glaucoma (Yoon et al., 2021). A case-control study found that patients with POAG had fewer natural teeth and a higher number of Streptococci oral bacteria compared to those without POAG (Polla et al., 2017). Additionally, recent tooth loss within the past two years has been linked to a 1.45-fold increased risk of POAG, with the strongest association observed in POAG subtypes with IOP <22 mmHg and early paracentral visual field loss (Pasquale et al., 2016).

Evidence also suggests a potential role for H. pylori infection in the development and progression of glaucoma. Histological confirmation of H. pylori infection has been found in 87.5 % of chronic OAG patients and 88.9 % of pseudoexfoliation glaucoma patients, while in 46.7 % of anemic control participants. This may suggest a shared susceptibility or that H. pylori is a potential cause for glaucoma development (Kountouras et al., 2001). Also, the aqueous humor samples of POAG and exfoliation glaucoma patients showed significantly higher mean concentration of anti-H. pylori-specific IgG compared to age-matched cataract control patients. Likewise, the serum concentration of anti-H. pylori antibodies was significantly greater in both POAG and exfoliation glaucoma patients compared to cataract controls (Kountouras et al., 2003). More recently, a robust link between the development of glaucoma and H. pylori infection has been confirmed (Doulberis et al., 2019) and after eradicating H. pylori, there was a favorable impact on the progression of POAG, resulting in decreased IOP and enhanced visual field parameters (Baim et al., 2019).

## 6 Microbial metabolites in glaucoma pathogenesis

Developing a therapeutic approach for neurodegenerative diseases through the manipulation of the microbiota is challenging. Therefore, comprehending the function of factors or substances released by the gut microbiome is essential. It may be more practical to focus on the progression of neurodegenerative diseases (Chen et al., 2023). Colony-forming bacteria release signals that affect immune regulation (McDermott and Huffnagle, 2014). The gut microbiota has an impact on specific immune cell functions in the retina due to the by-products of their metabolism (Scuderi et al., 2021). This influence is most noticeable in cases of glaucoma, particularly when analyzing the F/B ratio, a commonly used marker for intestinal balance.

A study conducted by Zhang et al. involved a metabolomic profiling of the cecum in rats with glaucoma revealed that there is a connection between an elevated F/B ratio and taurocholic acid, which is related to a reduction in the survival of retinal ganglion cells (Zhang et al., 2022). At the same time, a negative association was found between the F/B ratio and glutathione, which is an antioxidant that is linked with higher retinal ganglion cell survival (Zhang et al., 2022).

Gas chromatography/mass spectrometry is highly effective in examining metabolites from gut microbiota in animal models or patients with neurodegenerative conditions, such as POAG (Gong et al., 2020). The abundance of Megamonas and Enterobacteriaceae was lower in patients with POAG. Megamonas showed a positive correlation with citric acids and a negative correlation with L-g-glu-tamyl-L-alanine, hypoxanthine, and 3‑methoxy-4-hydroxyphenylglycol (Gong et al., 2020). Conversely, unidentified Enterobacteriaceae were found to have a negative correlation with citric acids and a positive correlation with 3‑methoxy-4-hydroxyphenylglycol (Gong et al., 2020). Moreover, the rise of E. coli in POAG patients implies that intestinal dysbiosis contributes to the development of the disease. Gram-negative bacteria, including E. coli, release lipopolysaccharide, which can trigger immune reactions and lead to heightened secretion of proinflammatory cytokines, nitric oxide, and eicosanoids. This sustained inflammation could potentially contribute to the pathogenesis of POAG (Lee et al., 2016). Moreover, patients with POAG have an increase in Prevotellaceae, which are butyrate producers (Gong et al., 2020) that exhibit anti-inflammatory effects (Schaefer et al., 2022). This butyrate may suppress NF-kB activation (Segain et al., 2000) and enhance regulatory T-cell differentiation (Furusawa et al., 2013). Studies have also shown differences in metabolites (glutamine, creatine, glycine, lysine, alanine, and hydroxyproline) between controls and POAG cases (Tang et al., 2022), as well as an increase in gut metabolite SCFAs (Chen et al., 2022b). SCFAs may be a biomarker for neurodegenerative diseases (Ruan et al., 2021) and can enhance neuroinflammation and retinal degeneration (Chen et al., 2022b).

## 7 Fecal microbiota transplantation as a recommended treatment for glaucoma

The link between bacterial populations, metabolites, and inflammatory pathways in retinal diseases suggests possible therapeutic approaches. These approaches could include increasing beneficial bacteria, reducing harmful bacteria, or introducing beneficial metabolites, and could also be targeted by pharmaceutical interventions. FMT operates through several crucial mechanisms, beginning with the transfer of a diverse population of beneficial microbes from a healthy donor into the gastrointestinal tract of a patient who has a dysbiotic microbiota at the compositional and functional levels. This new microbiota competes with and replaces pathogenic bacteria, which helps restore a healthy gut microbiome balance (Andary et al., 2024). By introducing the donor microbiota to the recipient, FMT promotes the reestablishment of healthy SCFA levels such as butyrate which is crucial for the modulation of the immune system, promoting gut barrier integrity, and reducing inflammation (Zhang et al., 2023; Andary et al., 2024).

In subsequent stages, the donor microbiota gradually colonizes and stabilizes within the recipient's gut. This interaction leads to the formation of a more diverse and robust microbial community, critical for proper digestive function, immune system modulation, and nutrient absorption, among other functions. The consistent interaction between the transplanted and the natural microbiota generates a long-lasting therapeutic effect (Zhang et al., 2023; Andary et al., 2024). Although this procedure is effective, the exact mechanisms of action are not yet fully understood. However, it appears that FMT works by promoting microbial diversity, establishing new microbiota, and modulating the immune system (Spindelboeck et al., 2021; Dahiya et al., 2023). Several studies have shown that FMT can help restore microbial diversity and effect metabolic functions by modulating the immune system (Spindelboeck et al., 2021; Dahiya et al., 2023). FMT also influences the dynamics of bacterial strains (Schmidt et al., 2022), affects bacteriophage populations (Łusiak-Szelachowska et al., 2020), and may even impact neurological (Li et al., 2024) and vascular diseases (Xu et al., 2023). Certain components of gut microbiota trigger the production of immune-modulatory compounds, helping regulate the immune response. Therefore, FMT can also impact the immune system. The combination of these factors contributes to the overall efficacy of FMT.

FMT is highly effective in treating Clostridium difficile infections (Dodin and Katz, 2014), resulting in the elimination of the infection and symptoms in 90 % of patients after a single treatment (Kassam et al., 2013). Moreover, FMT has been used for gastrointestinal tract autoimmune disorders (Anderson et al., 2012). Additionally, FMT has been applied in immune conditions outside the gastrointestinal tract, such as metabolic syndrome, multiple sclerosis, and idiopathic thrombocytopenic purpura (Borody et al., 2011a, b; Vrieze et al., 2012). However, it is undetermined whether FMT from healthy individuals can alleviate retinal ganglion cell loss and optic nerve damage in those with glaucoma.

Ocular diseases emerge from a reciprocal relationship between the eye and the gut, referred to as the gut–eye axis (Napolitano et al., 2021). The relationship involves the maintenance of retinal health through the regulation of the immune system and the production of anti-inflammatory factors. These include SCFAs, bacteriocins, secondary bile acids, indoles, and polyamines (Scuderi et al., 2021). In cases of dysbiosis, where pro-inflammatory bacteria proliferate at the expense of anti-inflammatory ones, this may lead to gut barrier disruption, metabolic endotoxemia, systemic inflammation, and retinal damage (Zysset-Burri et al., 2023). While the exact nature of the link between imbalances in the gut microbiota and eye diseases is not yet fully understood, it is believed that microbial dysbiosis at the ocular surface may lead to inflammation, which could further damage the eye and optic nerve. Several eye diseases, such as age-related macular degeneration, uveitis, diabetic retinopathy, dry eye, and glaucoma have all been linked to gut dysbiosis (Zinkernagel et al., 2017; Kalyana Chakravarthy et al., 2018; Lin, 2018; Gong et al., 2020; Huang et al., 2021; Zhang et al., 2022).

In the study by Chen et al., C57BL/6*J* mice were pre-treated with antibiotics and subsequently received fecal samples from glaucoma patients and healthy individuals. The mice that received the glaucoma samples exhibited increased retinal inflammation, leading to a loss of retinal cells (Chen et al., 2022a). Moreover, findings from a clinical trial involving 10 patients with Sjögren's syndrome, who received two fecal microbiota transplantations within a week, showed that their dry eye symptoms improved in 50 % of cases at a 3-month follow-up (Watane et al., 2022). The association between bacterial populations, metabolites, and inflammatory pathways in retinal diseases suggests possible therapeutic approaches. Therefore, using host microbiota-based therapies could be an additional treatment option for individuals with glaucoma, and we hypothesize that adding FMT to the standard treatment for glaucoma could be advantageous for those with this condition. However, it is essential to consider any harmful effects of FMT and any unforeseen interactions between FMT and conventional glaucoma treatment.

**Fig. 3:**
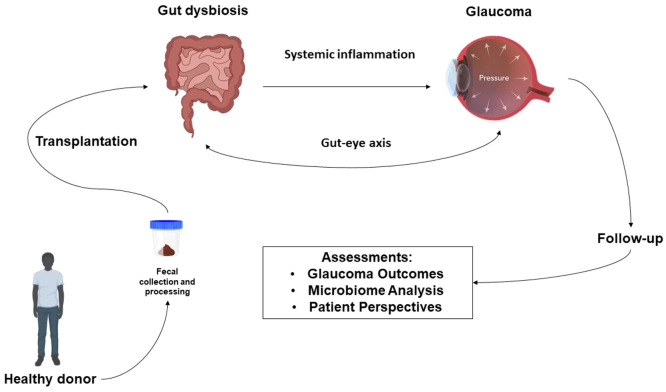
Schematic representation of fecal microbiota transplantation (FMT) as a potential therapeutic approach for glaucoma through the gut-eye axis. This figure illustrates the hypothesized role of FMT in managing glaucoma by addressing gut dysbiosis and its impact on the gut-eye axis. The process begins with the collection and processing of fecal material from a healthy donor, which is then transplanted into a patient with gut dysbiosis. The aim is to restore microbial balance, thereby reducing systemic inflammation that may contribute to increased intraocular pressure in glaucoma patients. Post-transplantation assessments are made to evaluate glaucoma outcomes, analyze changes in the gut microbiome, and gather patient feedback, with continuous follow-up to ensure treatment efficacy and safety. This diagram highlights FMT as a novel approach to potentially modulate glaucoma progression by influencing gut microbial composition and systemic health.

Qualitative studies, using patient interviews and surveys, could evaluate sociocultural factors that might affect the acceptance and adherence of patients to FMT. By understanding the impact of FMT on the microbiota, new insights into glaucoma pathogenesis and treatment responses might emerge. Assessing sociocultural factors is critical in ensuring patients' adherence and acceptance, particularly for the practical implementation of FMT among diverse populations. Microbiome analysis, clinical outcomes, and patient-reported data should be integrated to provide extensive insights into the possible applications of FMT in treating glaucoma and assessment of treatment response may involve monitoring intraocular pressure, visual field tests, and clinical symptoms. The gathered information could serve as a basis for developing clinical guidelines and policies for managing and treating glaucoma (Fig. 3).

### 7.1 Methods and dosage of FMT administration

To maximize the therapeutic benefits of FMT in glaucoma, it is crucial to critically evaluate the different methods and variables associated with FMT administration. FMT delivery varies based on the clinical context and can include upper endoscopy, nasoenteric tubes, or capsules for upper gastrointestinal tract administration, and colonoscopy, flexible sigmoidoscopy, or enemas for lower gastrointestinal tract administration (Fanizzi et al., 2024). The choice of route depends on the patient's condition and personal preferences (Wang et al., 2019). Methods for delivering FMT can target the upper, mid-, or lower gastrointestinal tract (Zhong et al., 2021). Each method has its specific pros and cons. Microbiota can be administered into the small intestine through oral capsules or a nasoduodenal tube, or into the large intestine via colonoscopy or enema (Long et al., 2018; Zhang et al., 2018). Conventional gastric delivery capsules and colon-targeted capsules are commonly used due to their low invasiveness and high patient acceptance, although they are costly and involve a significant capsule burden. Oral capsules with freeze-dried feces are effective, reducing the need for repeated clinic visits by both the donor and recipient. They are patient-friendly and risk-free in terms of procedure but face production challenges and need delayed-release formulations (Fanizzi et al., 2024). Similarly, fecal suspension infusion through colonoscopy or enema is also safe and effective. Colonoscopy allows direct bowel assessment but requires expertise and is costly. Enema is cost-effective and well-tolerated but unsuitable for patients with poor rectal sphincter tone. Rectosigmoidoscopy is less invasive and suitable for frail patients but is expensive. Additionally, FMT through a mid-gut transendoscopic enteral tube is a safe and convenient method that does not adversely affect patients' quality of life (Long et al., 2018). Naso-enteric tube delivery is cheap and does not need sedation but is uncomfortable and poses risks like vomiting. Upper endoscopy suits patients with severe colitis or those without an intact colon but is expensive, needs sedation, and has procedural risks. Of all the delivery methods, FMT administered via a nasoduodenal tube is frequently favored in clinical practice. This method requires minimal processing of fecal samples, allowing them to be used fresh on the day of donation. Typically, an intestinal lavage is performed beforehand. This technique maximizes the transfer of viable aerobic and anaerobic microbes, effectively reshaping the microbiota of both the small and large intestines.

Identifying the optimal route of administration for FMT is an important consideration for clinicians looking to incorporate this treatment into the care plan of patients with glaucoma. At present, it may not be possible to suggest a particular route for administration. FMT can be administered via both upper and lower gastrointestinal routes. However, some studies suggest that administering FMT through the upper gastrointestinal tract should be done carefully as it can lead to adverse events such as vomiting (Gweon et al., 2016) and aspiration pneumonia (Vermeire et al., 2016). This method has also drawbacks such as intubation discomfort and the inability to collect and analyze mucosal tissue samples. In addition to the effectiveness of the treatment, physicians consider various factors such as patient comfort and compliance, cost-effectiveness, invasiveness, risk of infection and aspiration, the need for multiple drugs, and relapse rate while choosing the administration route (Gulati et al., 2020). Physicians may decide on the appropriate dose, frequency, and duration of FMT for patients with glaucoma based on patient factors such as age, family history, severity of glaucoma, patient adherence, visual function, comorbidities, medical conditions, surgical history, and any medications the patient may be currently taking to ensure the safety and efficacy of the treatment. Existing guidelines on FMT for certain gastrointestinal disorders could be useful in informing their decision-making (Peery et al., 2024).

Studies have also used different dosages for FMT administration, such as introducing 250 or 500 ml of stool suspensions via colonoscopy (SahBandar et al., 2020; Boicean et al., 2022) or using capsules containing 30 g of stool (Serrano-Villar et al., 2021). The frequency of administration can range from a single session (Boicean et al., 2022) to multiple sessions (Chauhan et al., 2021). The intervals between sessions may vary from daily (Karolewska-Bochenek et al., 2021) to every four weeks (Chauhan et al., 2021). Currently, there is insufficient evidence to determine the effectiveness of FMT for treating glaucoma. Various aspects of the treatment such as the effective volume and dosage of FMT, frequency and duration of administration, and route of administration should be explored.

## 8 Evaluation of the theory

### 8.1 Potential efficacy of FMT

We have searched clinical trial databases such as Clinical-Trials.gov (https://clinicaltrials.gov/) and metaRegister of Controlled Trials (https://www.isrctn.com/) and have not found any current or upcoming trials that examine the use of FMT as an adjunct treatment option for individuals with glaucoma.

The impact of disrupted gut microbiome balance on various ocular disorders, such as dry eye (Bai et al., 2023), diabetic retinopathy (Thakur et al., 2022), uveitis (Kodati and Sen, 2019), keratitis (Jayasudha et al., 2018), orbitopathy (Biscarini et al., 2023), and age-related macular degeneration (Lima-Fontes et al., 2022) has garnered much interest among researchers and has become a trending research field. As far as we know, there is one clinical trial that used FMT for individuals with immune-mediated dry eye. The study investigated the efficacy and safety of FMT in treating immune-mediated dry eye in 10 patients; half of them had Sjögren's symptoms while the others showed early markers of it. These patients received two FMTs through enema, one week apart, from a single healthy donor. Within three months post-treatment, most of them reverted to their original microbiome, albeit some phyla, classes, and genera remained close to the donor's profile. The dry eye condition overall remained statistically unchanged following the therapy (Watane et al., 2022). Undoubtedly, despite some limitations such as a small sample size, short-term microbial composition similar to the donor, and subjective symptom improvement reporting, this study has advanced the use of FMT for treating autoimmune eye diseases. Most pertinent to dry eye, FMT has been applied in treating graft-versus-host disease, where dry eye plays a crucial role. Four patients with graft-versus-host disease received FMT treatment consisting of one or two doses of enemas taken one week apart in an open label study. The FMT treatment led to increased levels of beneficial bacterial strains such as *Bifidobacterium, Lactobacillus, Bacteroides*, and *Faecalibacterium* after four weeks. The patients experienced gradual improvement in gastrointestinal symptoms, such as defecation consistency and frequency (Kakihana et al., 2016). Additionally, recent findings propose that the relationship between gut microbiota and intestinal fungal species must be taken into account to optimize the effectiveness of FMT, as changes in the richness and diversity of gut fungal species were noted in uveitis patients when compared to controls (Jayasudha et al., 2019).

FMT has been effectively utilized for treating recurrent or refractory Clostridium difficile infections (Surawicz et al., 2013; Hui et al., 2019) and is currently being considered for primary Clostridium difficile infections (Juul et al., 2018), irritable bowel syndrome (El-Salhy et al., 2021), Crohn's disease (Fehily et al., 2021), obesity (Lai et al., 2018), chronic fatigue syndrome (Varesi et al., 2021), neurodegenerative diseases (Varesi et al., 2022), and neuropsychiatric disorders (Pascale et al., 2020). Studies have also demonstrated the effectiveness of FMT as a treatment option for other viral and bacterial infections such as norovirus infection (Barberio et al., 2020), cytomegalovirus colitis (Karolewska-Bochenek et al., 2021), and Carbapenem-resistant Enterobacteriaceae (Macareño-Castro et al., 2022). FMT brings about advantages, such as the restoration of balance of microorganisms (Wei et al., 2015), avoidance of leakage in the gut epithelial barrier (Zhao et al., 2021), an increase in SCFAs (Xiao et al., 2022), and a decrease in inflammation (Xiao et al., 2022). FMT might have the potential to improve the outcomes of age-related macular degeneration by reversing gut barrier disruptions and reducing inflammation affecting the retina (Parker et al., 2022). Due to these benefits, current research has centered on exploring its ability to influence the gut-eye axis (Gunardi et al., 2021) and many review articles have discussed and anticipated that FMT could be an effective treatment for extra-intestinal conditions, including ocular diseases (Baim et al., 2019; Fu et al., 2021; Hou et al., 2021).

### 8.2 Safety

There is limited clinical data on the safety of FMT in ocular disorders, but a clinical trial for immune-mediated dry eye found no negative adverse events in 10 patients who received FMT (Watane et al., 2022). FMT is generally considered safe for various conditions, but it is crucial to evaluate the risks and benefits for each patient and monitor closely for adverse events (Seon Young and Seo, 2021). Adverse events can vary based on the donor and recipient's health and administration route, with most being mild and gastrointestinal, but serious complications are possible including perforation, bacteremia, sepsis, multi-organ failure, and even death (Baxter and Colville, 2016; Janket et al., 2020), though they occur in <1 % of patients (Rapoport et al., 2022).

Marcella et al. reviewed the global incidence of FMT-related adverse events from 2000 to 2020, covering 129 studies with 4241 patients. They found FMT was generally well-tolerated, with some adverse events like diarrhea (10 %) and abdominal discomfort (7 %). Serious complications, including infections and fatalities, were reported in 1.4 % of patients (Marcella et al., 2021). The mortality rate for FMT was low (0.13 %), mainly due to aspiration pneumonia during or after the procedure. Microbiota-related deaths and serious adverse events were rare. Adverse events were more common in patients who had the procedure via upper gastrointestinal routes compared to lower routes (28.8 % vs. 17.5 %) (Marcella et al., 2021). Techniques like administering metoclopramide, upright positioning for mid-gut delivery, and adjusting infusion rates can reduce the risk of serious adverse events during upper gastrointestinal delivery of FMT. To evaluate long-term safety, the American Gastroenterology Association started the FMT National Registry, aiming to collect efficacy and safety data on 4000 patients over 10 years (The American Gastroenterological Association (AGA), 2020). To improve safety, China has been researching washed microbiota transplantation since 2014. Washed microbiota transplantation uses automatic filtration and washing, removing more viruses and pro-inflammatory mediators (Zhang et al., 2020). In 2020, the FMT-standardization Study Group released a consensus on washed microbiota transplantation methodology (Fecal Microbiota Transplantation-standardization Study Group, 2020) which offered guidance on the methodology of washed microbiota transplantation, distinguishing it from the manual FMT methods discussed in recent expert consensus and recommendations (Ng et al., 2020; Zhang et al., 2020).

### 8.3 Cost-effectiveness

FMT can be expensive due to the surgical nature of most procedures and strict donor inclusion criteria, limiting donor availability and increasing costs. Some facilities allow patients to select their own donors (Merenstein et al., 2014). In the US, the median range for outpatient costs related to severe OAG was $476 to $639, and the median glaucoma-related pharmacy costs ranged from $139 to $493 (Shih et al., 2021).

### 8.4 Availability and accessibility

The effectiveness of FMT relies on screening both the patient and the donor, with strict exclusion criteria for donor samples. Stool banks, which process and store stools for FMT, have been developed to screen donors and improve accessibility to treatment (Chen et al., 2021a). The importance of the relationship between the patient and the donor has also been explored, with some studies suggesting that anonymous donors may yield better results (Vindigni and Surawicz, 2017). The availability of FMT treatment for larger populations is expected to increase with the use of stool banks (Wynn et al., 2023).

### 8.5 Sociocultural competency

Major ethical challenges include ensuring donor privacy and obtaining informed consent from vulnerable groups, such as minority communities (Ma et al., 2017). Cultural and religious beliefs, dietary choices, and alcohol consumption can limit the availability of FMT in certain regions (Al-Bakri et al., 2021), and ethical concerns may arise when a vegan patient receives FMT from a non-vegan donor. These ethical and cultural issues may have implications for other applications of FMT, and eye diseases may have specific ethical considerations that should be taken into account for FMT treatment.

## 9 Conclusions

FMT could serve as an additional treatment option for glaucoma. Despite the difficulties of selecting appropriate donors and preparing samples, previous studies have shown that FMT can effectively treat various diseases. This highlights the potential significance of the gut microbiota in patients with glaucoma. Overall, FMT may be a valuable supplementary treatment for glaucoma and should be further evaluated through further research.

## Ethics approval and consent to participate

Not applicable.

## Consent for publication

Not applicable.

## CRediT authorship contribution statement

**Rasoul Ebrahimi:** Conceptualization, Data curation, Investigation, Methodology, Resources, Software, Writing – original draft, Writing – review & editing. **Yeganeh Farsi:** Resources, Data curation, Writing – original draft, Writing – review & editing. **Seyed Aria Nejadghaderi:** Conceptualization, Investigation, Project administration, Validation, Visualization, Supervision, Writing – original draft, Writing – review & editing.

## Declaration of competing interest

The authors declare that they have no known competing financial interests or personal relationships that could have appeared to influence the work reported in this paper.

## Data Availability

Data sharing not applicable to this article as no datasets were generated or analyzed during the current study.
